# Biomaterials: Chitosan and Collagen for Regenerative Medicine

**DOI:** 10.1155/2014/690485

**Published:** 2014-07-01

**Authors:** Yoshihiko Hayashi, Mitsuo Yamauchi, Se-Kwon Kim, Hideo Kusaoke

**Affiliations:** ^1^Department of Cariology, Nagasaki University Graduate School of Biomedical Sciences, Nagasaki 852-8588, Japan; ^2^Department of Oral Biology, NC Oral Health Institute, University of North Carolina, Chapel Hill, NC 27599-7455, USA; ^3^Department of Chemistry, Marine Bioprocess Research Center, Pukyong National University, Busan 608-737, Republic of Korea; ^4^Department of Environmental and Biological Chemistry, Faculty of Engineering, Fukui University of Technology, Fukui 910-8505, Japan

With contributions from USA, Taiwan, Japan, and Korea, this special issue holds great insight. This special issue offers comprehensive knowledge on chitosan and collagen as biomaterials, especially with respect to their basic biological and chemical properties, as well as clinical applications. Two review articles described the preparation and biological application of chitooligosaccharide and its derivatives and the relevance to clinical dentistry of distinct characteristics of mandibular bone collagen. Original articles reported seven experiments: 3 chitosan topics and 4 collagen topics. The former demonstrated the contributions for a proteomic view of chitosan nanoparticle to hepatic cells, the promotion of D-glucosamine to transfection efficiency, and chitin application as skin substitutes. The latter showed the contributions for hydroxyapatite-gelatin nanocomposite, genipin modification of dentin collagen, dentin phosphophoryn/collagen composite for dental biomaterial, and biological safety of fish collagen.

